# Supplementary material for: Semiconducting Polymer-Based Nanocomposite for Photothermal Elimination of Staphylococcus aureus Biofilm
Source: Microorganisms. 2025 Nov 11;13(11):2568. doi: 10.3390/microorganisms13112568 (PMC12654616; doi:10.3390/microorganisms13112568)
Supplement: Supplementary file 1 [file microorganisms-13-02568-s001.zip › microorganisms-3946216-supplementary.pdf]

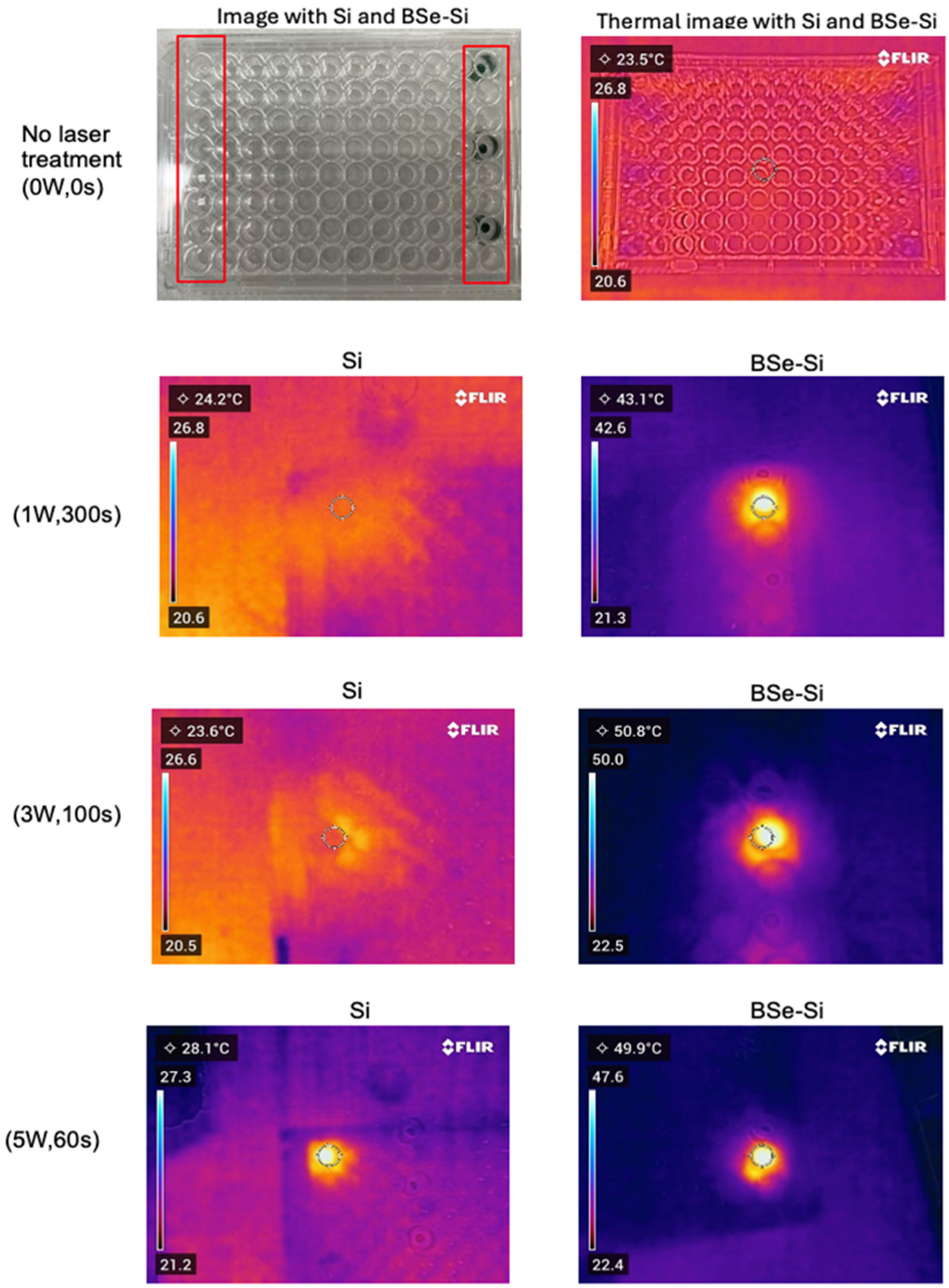

Figure S1. Thermal images of Si and BSE-Si disks at the end of laser exposure at 1W, 300s; 3W, 100s, and 5W, 60s.

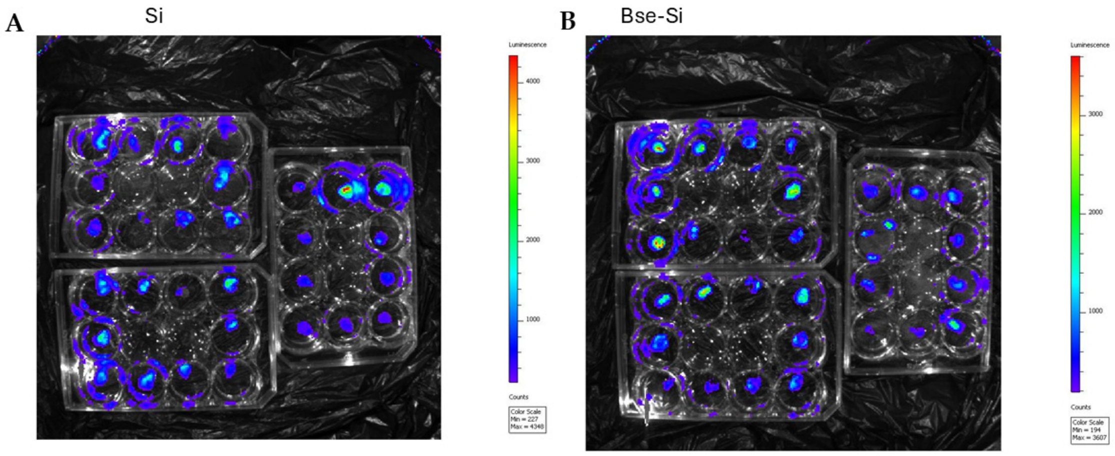

Figure S2. Bioluminescence of Xen 29 biofilms grown on Si (**A**) or BSe-Si (**B**) disks confirms metabolically active bacteria and uniformity of biofilms prior to implantation in mice.

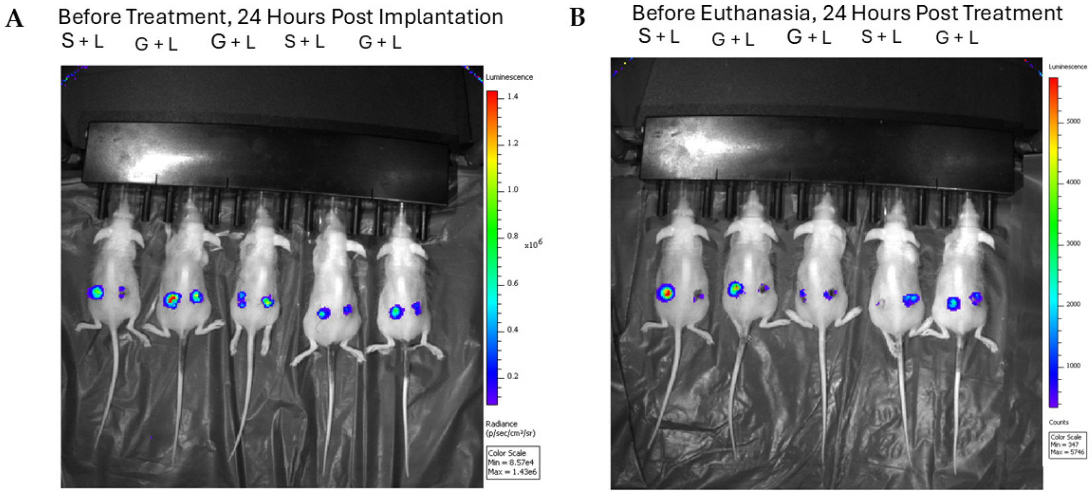

Figure S3. (**A**) Bioluminescence from Xen 29 biofilms on Si (left flanks) or BSe-Si (right flanks) 24 hr after disk implantation. (**B**) Bioluminescence 24 hr after PTT. S+L indicates treatment with systemic saline 1 hr before application of 3W or 800 nm light for 25 s over the disks in each flank. G+L indicates that mice received systemic gentamicin 1 hr before application of 3W or 800 nm light for 25 s over the disks in each flank.

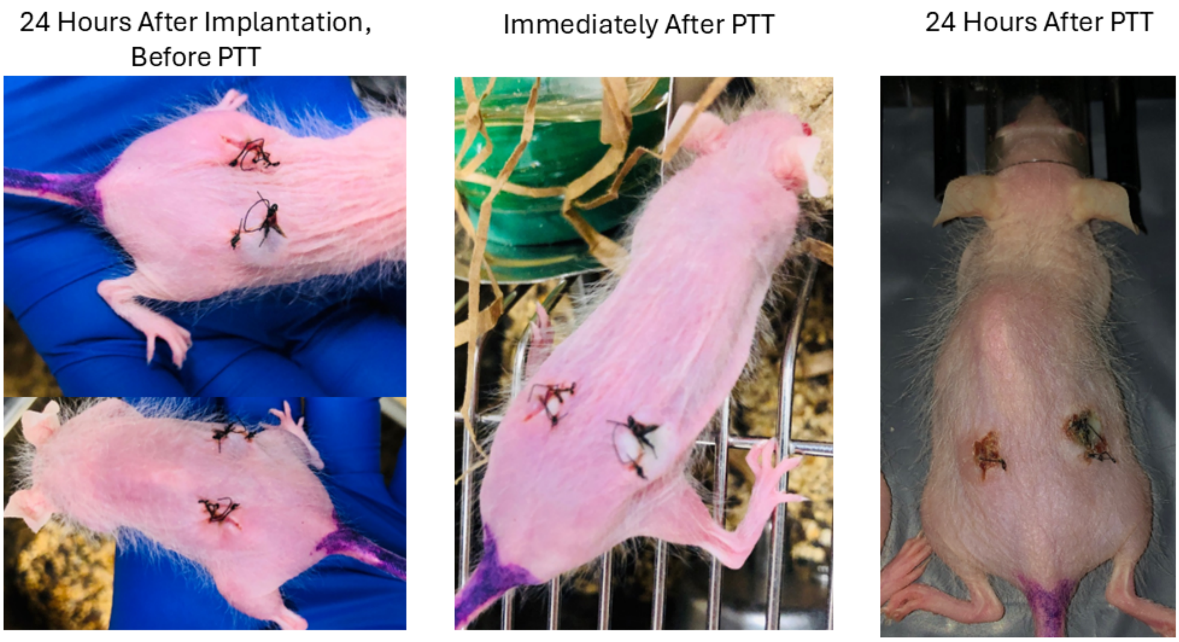

Figure S4. Images of a representative animal treated with gentamicin and PTT. Images show placement of the Si disk in the left flank, BSe-Si disk in the right flank and black suture for closure of the incision. The image taken immediately after PTT shows changes in skin color (blanching) over the BSe-Si disk, and to a lesser degree, over the Si disk. Image of the animal 24 hr after PTT shows skin changes indicative of a healing injury for both Si and BSe-Si disks. Protrusions under the skin also confirm that the disks remained at the site of their subcutaneous placement.

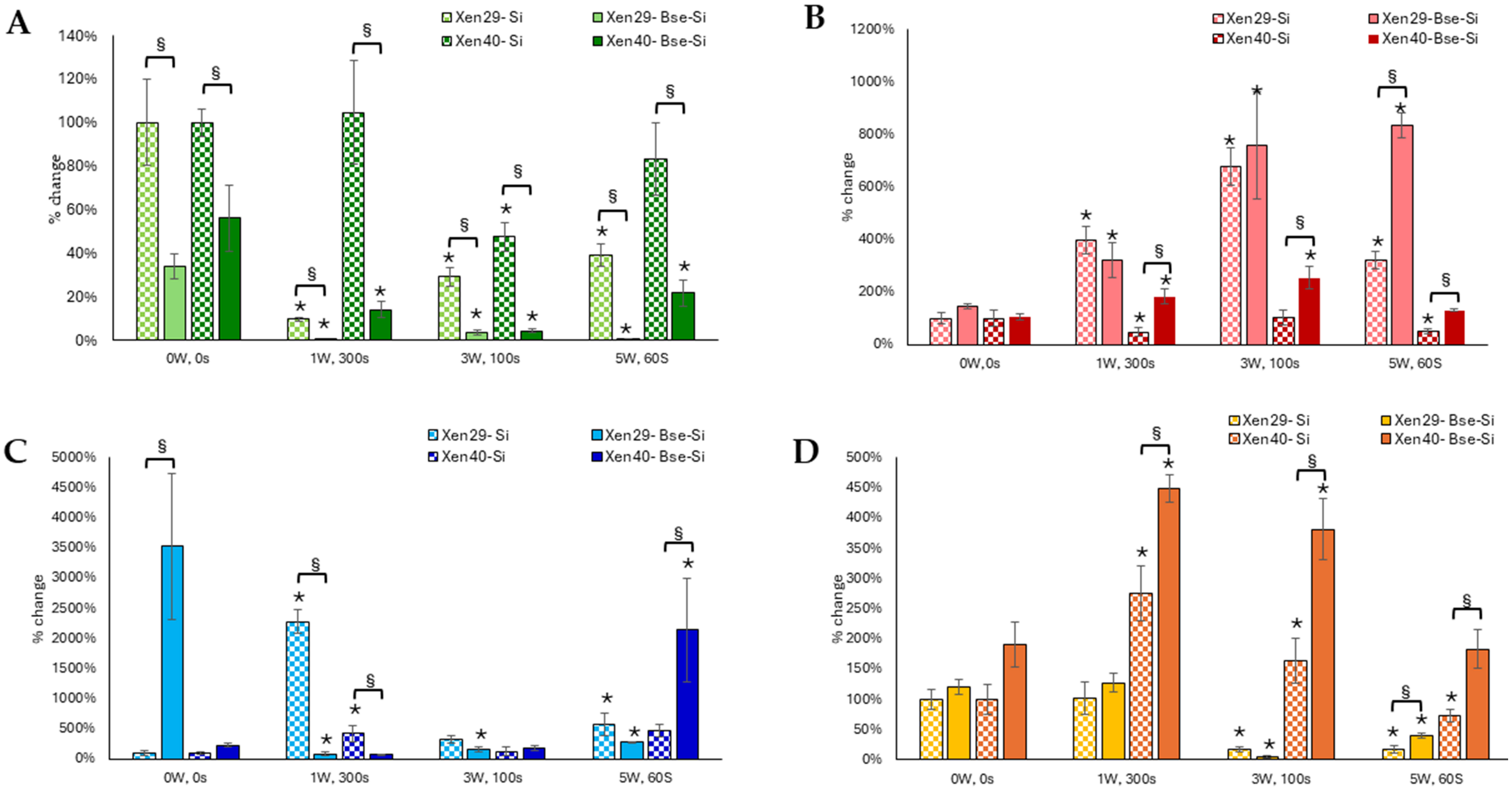

Figure S5. Percent change in fluorescence intensity of Xen 29 and Xen 40 biofilms, with fluorescence quantified from the microscopy images in Figure 8. (**A**) SYTO 9 (live bacteria). (**B**) Propidium iodide (dead bacteria). (**C**) Wheat germ agglutinin (polysaccharides). and (**D**) TOTO-3 (extracellular DNA). * denotes statistical significance compared to the 0W, 0s groups., with P<0.05. § indicates statistical significance between the two groups, with P<0.05
